# Temporal and spatial distribution of soil water and nitrate content affected by surface irrigation and fertilizer rate in silage corn fields

**DOI:** 10.1038/s41598-020-64876-7

**Published:** 2020-05-20

**Authors:** Yuchun Liu, Ning Wang, Changsong Jiang, Leigh Archer, Yao Wang

**Affiliations:** 1 Urban and Rural Construction College, Hebei Agricultural University, Hebei, 071001 China; 2 Horticultural Sciences Department, University of Florida, Southwest Florida Research and Education Center, Florida, FL 34142 USA

**Keywords:** Environmental impact, Hydrology

## Abstract

Among surface irrigation systems, long border and furrow are more adaptive to mechanized farming but may cause a non-uniform distribution of water and nutrients. In this study, field experiments were carried out in a flat silage corn field in Hebei, China to investigate the uniformity along the length of border or furrow to understand the spatial and temporal distribution characteristics of soil water, electrical conductivity, and nitrate. This will guide irrigation and fertigation management recommendations, land consolidation, and high standard farmland construction. Border and furrow irrigation were tested using fertilizer rates of 750, 600, 450 and 300 kg/ha. Low quarter distribution uniformity (DU_lq_) and storage efficiency (E) were quantified to determine the distribution of soil water and soil nitrate content. The results indicate heterogeneity along the length of the border or furrow is weak for soil water content and is moderate for nitrate content, based on the uniformity coefficient (CV). The average low quarter distribution uniformity of soil water (DU_lqW_) was 96.34, there was a significant effect of irrigation type on DU_lqW_, and the DU_lqW_ for border irrigation was 0.8% larger than that for furrow irrigation. The average low quarter distribution uniformity of nitrate content DU_lqN_ was 79.04, and there was no significant influence of irrigation type and fertilizer rate on DU_lqN_. Spatial and temporal distribution analysis showed that the variation of soil water in the 0–60 cm soil layer was larger than that in 60–100 cm soil layer, and the electrical conductivity (EC) and nitrate content gradually decreased with increasing soil depth. There was a decreasing trend in soil EC and nitrate content with decreasing fertilizer rates. The storage efficiency of water (E_W_) for border irrigation was 56.63, and significantly lower than that for furrow irrigation over the whole growth duration. The nitrate storage efficiency (E_N_) was 65.47, and there was no significant effect of irrigation type or fertilizer rate on E_N_. Even with longer borders or furrows of 90 m, the uniformity of water and nitrate along the length of the border or furrow is weak or medium, which can create non-uniform conditions for crop growth. Furrow irrigation may store slightly more water in the top 60 cm of soil compared to border irrigation. Fertilizer rate had no significant effect on the uniformity and distribution of soil water or nitrate.

## Introduction

According to the statistics of the Food and Agricultural Organization of the United Nations, globally around 86% of the area equipped for full control irrigation is surface irrigation. Surface irrigation adapts well to most crops, low to medium soil infiltration characteristics and crop mechanization as well as environmental conditions^1^. Surface irrigation delivers water to crops using a gravity-fed, overland flow of water, which may result in non-uniform distribution of water, excessive percolation losses near the outlet due to more infiltration opportunity time, some areas remaining under-watered or over-watered^2^. Irrigation efficiency is the main consideration in the design and management of surface irrigation systems^3,4^. With surface irrigation, water distribution can be highly variable along the border or furrow length, this “down field” non-uniformity is usually combined with “inter-row” non-uniformity of water and nitrogen fertilizer distribution^5^. Early in the 1990s, distribution uniformity and irrigation efficiency were first analyzed in detail^6,7^, and in recent years, technological development in aerial imaging can automatically measure distribution uniformity^8^.

Non-uniform distribution and low irrigation efficiency may have effects on crop yield^9^, crop water requirement^10^, crop water use efficiency^11^, deep drainage^12^, nitrate leaching, and soil nitrate content^13^. Along with irrigation efficiency, fertilizer uniformity is an evaluation criterion for irrigation and fertilizer application performance. One study on the effect of sprinkler uniformity on nitrate leaching and soil nitrate content in a carrot field suggests that sprinkler irrigation non-uniformity has little impact on nitrate movement^13^. There was less study on the uniformity of surface irrigation compared to sprinkler irrigation. For surface irrigation, low irrigation efficiency may be due to poor design or irrigation control though irrigation efficiency can be increased with management strategies. When the cutoff time is held constant, simulations with SIRMOD software showed that cutback and surge irrigation methods were able to increase irrigation efficiency 11.66% and 28.37% respectively, farmers can identify the proper input discharge to achieve a maximum of irrigation efficiency based on inflow regime choice^14^. By applying optimal irrigation, application efficiency in the treatment of a row plant on a ridge of 37.5 cm(S_0_) increases by up to 9% and two rows plant on a ridge of 75 cm treatment(SR2) increases by up to 15% higher than the conventional irrigation^15^.

Field layout, including the number of border stripes, length of borders or furrows, the spacing of furrows, etc. are additional factors that influence the distribution uniformity and application efficiency of irrigation water. These elements affect the efficiency of agricultural machinery and are critical design factors in land consolidation and high standard farmland construction. An Indus Basin irrigation project in Pakistan^16^ found the optimal field layout to maximize irrigation performance, the application of 50 mm irrigation water was recommended and achieved a low quarter distribution uniformity of 0.75 and an application efficiency of 80% under clay loam soil. In the North China Plain^17,18^ 30 m furrow in silt loam soil was adopted in cotton experiments to evaluate the distribution uniformity of soil water, nitrate content, and the growth and yield of cotton along the length of furrow, results suggest the uniformity of soil water, nitrate content, and ammonium content along furrow belonged to medium variability, while cotton yield was more uniform. In Karnal District, Haryana, India^2^, 50 m border and furrow systems in clay loam soil was evaluated to determine the effect of inflow type on distribution uniformity and application efficiency, results suggest that water distribution was uniform and improved application efficiency was evident using multi-outlet pipe compared to a single-outlet pipe. In Iran^19^, the uniformity of nitrate fertigation in different soil textures in 100~200 m furrow irrigation systems were evaluated, fertilizer injection in the first half and during the entire irrigation event was better in terms of distribution uniformity and infiltrated fertilizer, and no significant difference was observed between the distribution uniformity of free-drainage and blocked-end experiments. To increase the application efficiency and distribution uniformity and decrease fertilizer loss of furrow irrigation in sloping fields, meandering furrow irrigation was experimented and suggested t be adopted^20^.

The numerical model provides a different method to understand knowledge on the migration law of soil water and solute under surface irrigation. SURDEV software was used to evaluate the existing border systems and to optimize border dimensions, this software suggests alternatives for existing irrigation systems to achieve more than 60% application efficiency. Optimizing border dimensions is a practical way to maximize water resources use efficiency, a border length of 60–150 m and a border width ranging from 4–65 m was optimized for different flow rates in light and medium soils^21^. A simulation-optimization model^22^ was developed to optimize the design and management of alternate-furrow and conventional furrow fertigation, and the model could also minimize both water and nitrate losses for all irrigation treatments with acceptable distribution uniformity, the result of the model showed that alternate-furrow irrigation could strongly increase water and nitrate application efficiency as compared with conventional furrow irrigation. The software SIRMOD was used to evaluate border irrigation systems under open- and closed- end conditions, and open-end conditions improved recession time over closed-end system^23^. Soil heterogeneity may result in high variation in hydraulic properties of soil, so two-dimensional stochastic finite element models were developed for simulating soil water flow^24^ and contaminant transport^25^ through soils under furrow irrigation with the main focus being on the incorporation of the effects of soil heterogeneity, and the simulation of the models showed that because the influence of the variability of the properties of soil and effects of parameter hysteresis on water flow and water content redistribution were considered, better agreement with experimental measurements in comparison with a deterministic model^24^, and soil heterogeneity causes an enhancement in the solute spread in all directions and in the case of water flow in direction parallel to soil layering, the enhancement in transverse direction of mean flow is higher than the one in longitudinal direction^25^. Though field experiments are time and labor-consuming, experiment data are needed to evaluate the irrigation efficiency using local conditions and to calibrate the simulation model.

The North China plain, research district of this study, is short of water resources, but border irrigation is still widely used in the crop production, and suitable water-saving irrigation technologies, like furrow irrigation, drip irrigation, are suggested to be adopted in the winter wheat and corn production in the North China Plain. When furrow irrigation was introduced to the farmers, the possible problem of low irrigation uniformity was put forward. Long border or furrow systems are better adapted to mechanized farming but may cause the non-uniform distribution of water and fertilizers. The problem of more fertilizer used is another distress in crop production in the North China Plain. Border and furrow irrigation used in silage corn production with standard farm management under different fertilizer rates were analyzed in this study to (i) evaluate the uniformity and distribution of soil water, electrical conductivity, and nitrate in different soil depth along a long border or furrow (90 m) during the whole growth period of silage corn; and (ii) analyze the effects of irrigation type and fertilizer rate on the distribution uniformity and the spatial and temporal distribution of soil water, electrical conductivity and nitrate content. The results of the study can provide theoretical and technical support for the water and fertilizer management in crop production in the North China plain.

## Materials and Methods

### Experiment site

**Figure 1:**
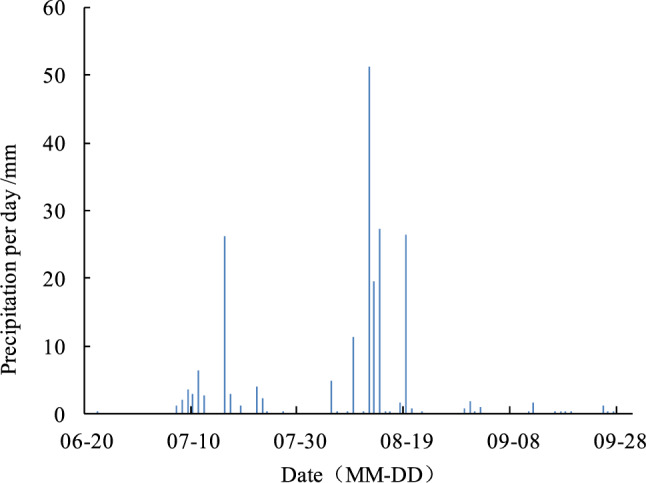
The precipitation during the experiment.

Field experiments were conducted in 2018 at the Nanlonggui village, Luquan District, Shijiazhuang, Hebei, China. The study area is flat and located at 37°56′54.1″N, 114°25′49.6″E and 122 m above the mean sea level, with semi-humid continental monsoon climate. Yearly average air temperature, sunshine duration, precipitation, and evaporation of the region are 12.2 °C, 2554 h, 536 mm and 1610.1 mm respectively. The soil is loam throughout the top 100 cm. The physical and hydraulic properties of the soil are presented in Table 1. The initial values of organic matter, rapidly available phosphorus and rapidly available potassium were 2%, 11.89 mg/kg and 158 mg/kg, respectively. Silage corn was planted on June 27 and the experiment period was 95 d. The precipitation during the experiment was 206.9 mm (Fig. 1), and the total precipitation in 2018 was 509.2 mm, which is typical for the region.

**Table 1:** Physical and hydraulic properties of experimental soils.

Soil Depth (cm)	Bulk density (g. cm^−3^)	Soil particle grading (USA)			Texture	Saturated water content (cm^3^ cm^−3^)	Field capacity (cm^3^ cm^−3^)
		Sand/%	Silt/%	Clay/%			
00∼20	1.25	47.42	50.38	2.2	Loam	0.302	0.147
20∼40	1.33	38.47	58.98	2.55	Silty loam	0.39	0.174
40∼60	1.33	43.09	56.33	2.98	Clay loam	0.365	0.182
On 60∼80	1.29	40.69	54.23	2.68	Silty clay loam	0.418	0.189
80∼100	1.43	40.6	56.6	2.8	Clay loam	0.432	0.250

### Experimental design

Irrigation type and fertilizer rate were considered in the experiments. Border irrigation and furrow irrigation were used for irrigation. Fertilizer rates were 750, 600, 450 and 300 kg/ha, based on grower’s standard rates and experimental rates for decreasing fertilizer application amount in China. There were 8 experimental treatments, 3 replications for each treatment, for a total of 24 experiments plots.

The borers were 90 m by 4.8 m with 9 rows of silage corn planted within each border. The width of the border was determined by the standard widths of common agricultural machinery, including seeders, pesticide applicators, and harvesters. The length of the furrows was 90 m with a spacing of 60 cm. The width at the top and bottom of the furrow was 40 cm and 20 cm respectively, and the depth of the furrow was 20 cm. The furrows were constructed with a ditcher and artificially shaped. There were 8 furrows in each experiment plot. Both the experimental borders and furrows were closed-end.

The experimental plots were laid out in a randomized complete block design. The corn rows were spaced 60 cm apart and the distance between the plants cultivated along the lines was 30 cm. The corn density was 78 thousand plants ha^−1^, which is a typical grower standard. Plots were separated by 1.5 m spaces to ensure that the treatments were independent of each other. The plots were regularly weeded and treated for pests and diseases during the growth period. The soil preparation of the experiment field was carried out before sowing using laser leveling (1JP350), and the slope of the soil preparation was 0.5‰. The variety of the silage corn used in the experiments was Keyu 188, which is a variety commonly grown in the region.

The flow rate of the well for irrigation was 35 m^3^/h, only one border or plot was irrigated for border irrigation and the unit flow per meter width was 2.03 L/s. 8 furrows were irrigated together for furrow irrigation and the stream size per furrow was 1.22 L/s. Due to soil water content at sowing, the field was irrigated 70 mm immediately after sowing on June 28 to maximize the emergence rate. No other irrigation was required for the duration of the experiment due to adequate precipitation. A compound fertilizer (28%N, 6%P_2_O_5_ and 6%K_2_O) and silage corn seeds were applied at the same time with a seeder at a time, and no other fertilizer was applied for the extent of the experiment.

### Measurement methods and calculations

Before sowing, on June 27, soil samples were taken using a soil drilling method at three randomly selected points at depths of 0–20, 20–40, 40–60, 60–80 and 80–100 cm intervals to measure the initial soil water content, electric conductivity, and nitrate content. During the growth period, soil samples were taken every 8 days. Additional samples were taken after irrigation and precipitation, for a total of 12 soil samples throughout the experiment. 4 sections along the length of border or furrow: 9, 33, 57 and 81 m from the water inlet were selected for samples. The soil samples were taken between corn plants within the corn rows. Rows with proper density and uniform growth were selected for samples.

From these soil samples, a portion taken was to measure soil water content using the oven method. The rest was air-dried, milled and passed through a 2 mm sieve. Soil extract was prepared by mixing soil and water in 1:5 ratio (5 g soil and 25 mL water), the sample was allowed to settle for 5 minutes and filtered using filter paper and funnel. The Zhengda ADS-us/cm pen-type waterproof conductivity detector was used to measure electrical conductivity, the Leici PXSJ-226 ion meter was used to measure nitrate content of the soil extract. The nitrate content of the soil extract was converted into that of the soil.

For each soil depth and at different growth stages, the coefficient of variation (CV) was used for quantifying the temporal and spatial variation of the water content, electrical conductivity and nitrogen along the length of the border and furrow. Heterogeneity was considered weak when CV ≤ 0.1, moderate when 0.1 < CV ≤ 1 and strong when CV > 1^26^.

Distribution uniformity (DU) in irrigation is a measure of how evenly water is applied to an area, the most common measure of DU is the low quarter distribution uniformity (DU_lq_)^4,6^, which is the measure used for this study. The low quarter distribution uniformity of soil water (DU_lqW_) and nitrate content (DU_lqN_) was calculated as:

1 $${{\rm{DU}}}_{lqW}={{\rm{W}}}_{lq}/{{\rm{W}}}_{avg}\ast 100 \% $$

2 $${{\rm{DU}}}_{lqN}={{\rm{N}}}_{lq}/{{\rm{N}}}_{avg}\ast 100 \% $$

where, DU_lqW_ is the low quarter distribution uniformity of soil water, W_lq_ is the water amount in the top 0–100 cm of soil when soil water content is the low quarter value along the length of border or furrow, W_avg_ is the total accumulated soil water amount in the top 0–100 cm of soil when soil water content is the average value along the length of border or furrow. DU_lqN_ is the low quarter distribution uniformity of nitrate content, N_lq_ is the nitrate amount in the top 0–100 cm of soil when soil nitrate content is the low quarter value along the length of border or furrow, N_avg_ is the total accumulated soil nitrate amount in the top 0–100 cm of soil when soil nitrate content is the average value along the length of border or furrow.

Soil storage efficiency of soil water and nitrate content was used to quantify the distribution of water and nitrate. Soil water storage efficiency (E_w_) and soil nitrate storage efficiency (E_N_) were calculated as^27^:

3 $${{\rm{E}}}_{w}=\frac{{{\rm{W}}}_{60}}{{{\rm{W}}}_{100}}\ast 100 \% $$

4 $${{\rm{E}}}_{N}=\frac{{{\rm{N}}}_{60}}{{{\rm{N}}}_{100}}\ast 100 \% $$

W_60_ is the soil water amount in the top of 0–60 cm of soil, which is where a majority of corn roots are located. W_100_ is the cumulated soil water amount in the top of 0–100 cm of soil. N_60_ is the soil nitrate amount in the top 0–60 cm of soil. N_100_ is the cumulated soil nitrate amount in the top 0–100 cm of soil. A higher value of E_*w*_ and E_*N*_ indicates more water and nitrate stored in the root zone and enhances growth.

### Statistical analysis

Correlation analysis among the spatial and temporal distribution of the soil water content, electrical conductivity, and nitrate content was examined to explore the correlation between water EC and nitrate distribution.

## Results

### The uniformity of soil water, electric conductivity, and nitrate

**Figure 2:**
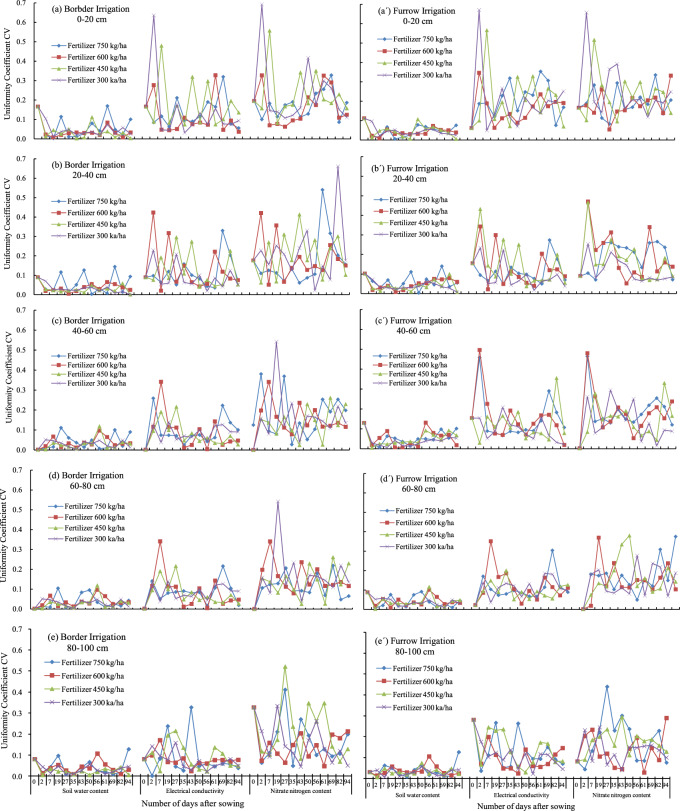
The uniformity coefficient *CV* of soil water, electrical conductivity and nitrate content along the border or furrow length at a soil depth of 0–20, 20–40, 40–60, 60–80 and 80–100 cm for fertilizer rates of 750, 600, 450 and 300 kg/ha under border and furrow irrigation.

The CV of soil water, electrical conductivity, and nitrate along the border or furrow length are shown in Fig. 2. The CV of soil water is 0.001~0.170 and 0.003~0.140, and the average value is 0.038 and 0.044 for border and furrow irrigation respectively, heterogeneity of soil water along the length of border or furrow is approaching weak. The average CV of soil water for the soil of 0–20, 20–40, 40–60, 60–80 and 80–100 cm is 0.039, 0.032, 0.027, 0.025 and 0.029 for border irrigation, and 0.038, 0.039, 0.040, 0.033 and 0.027 for furrow irrigation respectively. The heterogeneity of soil water in the upper 0–60 cm is slightly greater than at a depth of 60–100 cm, and that for furrow irrigation is slightly greater than that for border irrigation. There is no obvious difference in the CV of soil water during the experimental period of silage corn or for different fertilizer rates.

The CV of electrical conductivity is 0.001~0.636 and 0.017~0.671, and the average value is 0.101 and 0.135 for border and furrow irrigation respectively, indicating moderate heterogeneity of electrical conductivity along length of border or furrow. The average CV of electrical conductivity for soil layers of 0–20, 20–40, 40–60, 60–80 and 80–100 cm is 0.111, 0.092, 0.070, 0.065 and 0.067 for border irrigation, and 0.153, 0.097, 0.105, 0.086 and 0.098 for furrow irrigation respectively. Heterogeneity of electrical conductivity is moderate in the top 0–20 cm for border irrigation and both 0–20 and 40–60 cm for furrow irrigation and is weak in the other soil layers. The CV of electrical conductivity is 0.132 from Jun 29 to July 27 and 0.121 from August 1 to September 29 for border irrigation, compared to 0.162 from June 29 to July 27 and 0.090 from August 1 to September 29 for furrow irrigation. The heterogeneity of electrical conductivity is slightly larger for the early stage of the growth period than that for the late growth stage. There is no difference in uniformity coefficient CV of electrical conductivity for different fertilizer rates.

The CV of nitrate content is 0.000~0.692 and 0.001~0.657, with an average value of 0.176 and 0.169 for border and furrow irrigation respectively, Heterogeneity of nitrate content along the length of border or furrow is moderate. The average CV of nitrate content for soil layers of 0–20, 20–40, 40–60, 60–80 and 80–100 cm is 0.160, 0.155, 0.137, 0.110 and 0.142 for border irrigation, and 0.172, 0.133, 0.126, 0.121 and 0.123 for furrow irrigation respectively. Heterogeneity of soil nitrate content is moderate at each soil depth. The CV of nitrate was 0.185 from Jun 29 to July 27 and 0.172 from August 1 to September 29 for border irrigation, compared to 0.201 from June 29 to July 27 and 0.166 from August 1 to September 29 for furrow irrigation. The results indicate heterogeneity of nitrate is slightly greater for the early stage of the growth period than that for the late growth stage. There was no visible difference in the uniformity coefficient CV of electrical conductivity for different fertilizer rates.

As irrigation and fertilizer were applied one time during the whole season in this study, precipitation probably had an important role in the distribution of soil water and nitrate content than irrigation and fertilizer. The CV of soil water, electrical conductivity and nitrate on July 16 and on August 16 after precipitation 26.3 and 98 mm respectively were analyzed. The results showed that the CV of soil water tends to increase after precipitation especially in the shallower soil layer 0~40 cm and in the deeper soil layer of 60~100 cm, and the CV of electrical conductivity and nitrate tends to increase after precipitation in the deeper soil layer of 60~100 cm. The redistribution of soil water after precipitation may cause the change of the CV of soil water, electrical conductivity and nitrate content in the soil layer.

DU_lqW_ and DU_lqN_ are shown in Table 2. The average DU_lqW_ was 96.22% and 95.50% for border and furrow irrigation respectively over 90-day trial. Analysis of variance suggests that irrigation types had a significant effect on DU_lqW_. The DU_lqW_ for border irrigation was 0.8% larger than that for furrow irrigation at the 0.01level. Irrigation type had no effect on DU_lqW_ after 70 cm irrigation on June 29. The DU_lqW_ over the whole growth period was 95.07%, 95.68%, 96.29% and 96.39% for fertilizer rates of 750, 600, 450 and 300 kg/ha respectively. DU_lqW_ was gradually increased with a decrease in fertilizer rate. After 70 cm irrigation on June 29, the DU_lqW_ was 97.41%, 98.22%, 98.72% and 92.92% for fertilizer rates of 750, 600, 450 and 300 kg/ha respectively, and was larger than the average DU_lqW_ over the whole growth period. The DU_lqN_ was 81.45% over the whole growth period and 76.64% after 70 cm irrigation on June 29. There was no obvious effect of irrigation type and fertilize rate on DU_lqN_.

**Table 2:** Analysis of variance for low quarter distribution uniformity of soil water and nitrate.

Irrigation type	Fertilizer Rate/kg/ha	Average over the whole period		Value in June 29^a^	
		DU_lqW_/%	DU_lqN_/%	DU_lqW_/%	DU_lqN_/%
Border irrigation	750	95.29	84.13	98.31	88.24
	600	95.95	83.05	98.19	69.40
	450	96.92	79.06	98.71	77.28
	300	96.73	78.86	92.52	72.15
Furrow irrigation	750	94.86	80.49	96.52	83.93
	600	95.42	81.64	98.24	68.50
	450	95.66	81.78	98.74	86.15
	300	96.04	82.55	93.32	67.48
P-Value, Analysis of variance	Fertilizer amount	0.041*	0.792	0.014*	0.067
	Irrigation type	0.030*	0.856	0.709	0.941

Note: **Significant at the 0.01 level, *Significant at the 0.05 level, ^a^The low quarter distribution uniformity after 70 mm irrigation on June 28.

### Distribution of water, electric conductivity, and nitrate

**Figure 3:**
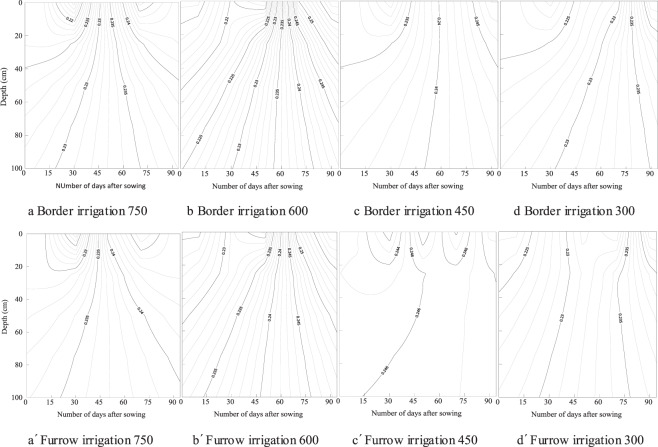
The spatial and temporal distribution of soil water content (g/g) in the top 0–100 cm of soil over 90 days for fertilizer rates of 750, 600, 450 and 300 kg/ha under border and furrow irrigation.

Temporal and spatial distribution of soil water content in the soil layer is shown in Fig. 3. Soil water was low at the beginning of the growth period because of the low initial soil water content, but gradually increased with irrigation and precipitation, reached the maximum 75 days after sowing, and gradually decreased at the end of the growth period. The soil water content in 0–60 cm changes from 0.024~0.238, 0.024~0.249, and the average value is 0.162~0.173, 0.166~0.176 for border and furrow irrigation respectively. The soil water content in 60–100 cm soil layer changes from 0.163~0.248 and 0.162~0.259, and the average value is 0.185~0.202, 0.187~0.203 for border and furrow irrigation respectively. The variation range of soil water in the 0–60 cm soil layer is larger than that in the 60–100 cm soil layer. The deeper soil water content is greater through the whole growth duration. There was no effect of irrigation type and fertilizer rate on the spatial and temporal distribution of soil water content.

**Figure 4:**
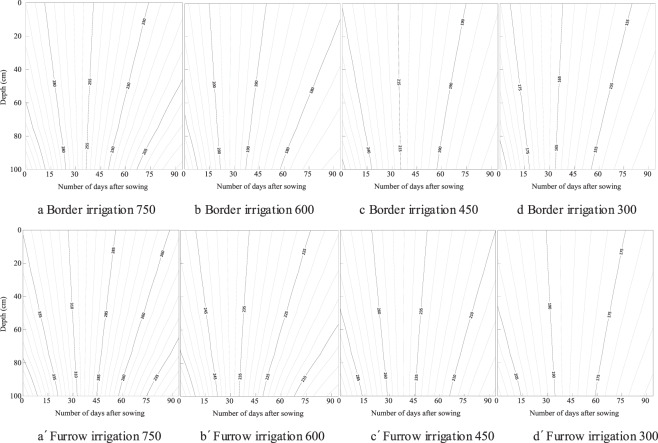
The spatial and temporal distribution of soil electrical conductivity (µs/cm) in the top 0–100 cm of soil over 90 days for fertilizer rates of 750, 600, 450 and 300 kg/ha under border and furrow irrigation.

The spatial and temporal distribution of EC is shown in Fig. 4. Over the growth period, EC gradually decreased. The EC in the 0–20 cm soil layer decreased from 245 to 220 µs/cm in the furrow irrigation treatment with a fertilizer rate of 600 kg/ha. The soil EC obviously decreased with increasing soil depth. The soil layer of 0–20, 20–40, 40–60, 60–80 and 80–100 cm for the border treatment with fertilizer rate of 750 kg/ha, the average EC was 250.5, 210.4, 192.5, 186.5 and 184.6 µs/cm respectively. There was a significant effect of fertilizer rate on the spatial distribution of EC. The soil EC decreased with the decreasing of fertilizer rates. In the top 0–20 cm, the average EC per measurement date was 250.5, 239.1, 240.2 and 219.4µs/cm for border irrigation, and was 250.9, 245.5, 244.9 and 225.7 µs/cm for furrow irrigation respectively. The average EC content for furrow irrigation was 1.9% and 10.8% larger than that for border irrigation at 0–20 and 20–40 cm soil respectively.

**Figure 5:**
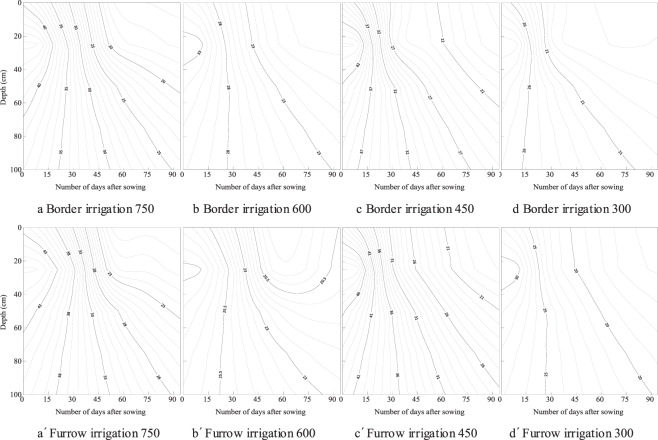
The spatial and temporal distribution of soil nitrate content (mg/kg) in the top 0–100 cm of soil over 90 days for fertilizer rates of 750, 600, 450 and 300 kg/ha under border and furrow irrigation.

The spatial and temporal distribution of nitrate is shown in Fig. 5. The same distribution characteristics were found for nitrate and EC. Soil nitrate content gradually decreased from the beginning to the end of the growth period. For furrow irrigation at a fertilizer rate of 600 kg/ha, soil nitrate in the top 0–20 cm decreased from 28 to 20 mg/kg. Soil nitrate clearly decreased with increasing soil depth. The average nitrate was 23.6, 20.3, 18.3, 17.4 and 17.2 mg/kg for the soil layer of 0–20, 20–40, 40–60, 60–80 and 80–100 cm respectively for border irrigation with a fertilizer rate of 750 kg/ha. There was a significant effect of fertilizer rate on the spatial distribution of nitrate in the soil layer. Soil nitrate decreased with lower fertilizer rates. In the top of 0–20 cm, for example, the average nitrate was 23.6, 22.8, 22.9 and 19.5 µs/cm for border irrigation, and was 23.6, 23.2, 22.9 and 20.6 µs/cm for furrow irrigation respectively. The average nitrate content for furrow irrigation was 1.8% and 8.7% larger at 0–20 and 20–40 cm than that of border irrigation.

Correlation analysis for soil water content, electrical conductivity, and nitrate was examined based on the spatial and temporal distribution of each of these parameters. The results are listed in Table 3. It can be suggested that the correlation between soil water content and electrical conductivity or nitrate is weak, while the correlation between electrical conductivity and nitrate is significant at the 0.01 level. The regression equation between nitrate and electrical conductivity for the experimental field is expressed as:

5 $${\rm{y}}=0.0876x+1.2595$$

where y is the soil nitrate content (mg/kg), x is the soil electrical conductivity (us/cm), and the correlation coefficient of the equation is 0.9200.

**Table 3:** Correlation among the uniformity coefficient, the distribution of soil water, soil electric conductivity (EC), and soil nitrate content(NN) under border and furrow irrigation at the fertilizer rate of 750, 600, 450 and 300 kg/ha.

Item	Fertilizer amount kg/ha	Border Irrigation			Furrow Irrigation		
		Water-EC	Water-NN	EC-NN	Water-EC	Water-NN	EC-NN
Uniformity of Soil water, Electricity Conductivity, Nitrate (NN)	750	0.252*	0.109	0.419**	0.014	−0.216	0.455**
	600	0.215	0.22	0.668**	−0.054	−0.187	0.587**
	450	0.082	0.063	0.559**	0.033	−0.131	0.676**
	300	0.467**	0.282*	0.598**	−0.01	0.152	0.729**
Distribution of Soil water, Electricity Conductivity, Nitrate	750	0.098	0.063	0.959**	0.153	0.155	0.926**
	600	0.124	0.106	0.903**	0.194	0.149	0.927**
	450	0.157	0.153	0.919**	0.219	0.199	0.938**
	300	0.195	0.122	0.812**	0.242	0.214	0.897**

Note: N = 65, **Correlation is significant at the 0.01 level, *Correlation is significant at the 0.05 level.

Results for water and nitrate storage efficiency in the soil layer are listed in Table 4. The average E_W_ was 56.32% and 56.93% for border and furrow irrigation respectively. E_W_ after 70 cm irrigation on June 29 was 56.88% and 57.65% for border and furrow irrigation. An analysis of variance suggested that irrigation type had a significant effect on E_W._ E_W_ for border irrigation was 1.1% and 1.3% lower than that for furrow irrigation through the trial and after 70 mm irrigation on June 29 respectively, which is significant at the 0.05 level. E_W_ was significantly lower at a fertilizer rate of 750 and 600 kg/ha than at a rate of 450 and 300 kg/ha after the 70 mm irrigation on June 29 at the 0.01level. The nitrate storage efficiency E_N_ was averaged 62.13% during the trial and 68.80% after 70 cm irrigation on June 29 respectively. There were no significant effects of irrigation type or fertilizer rate on nitrate storage efficiency.

**Table 4:** The storage efficiency of soil water and nitrate content and the results of the analysis of variance.

Irrigation type	Fertilizer Rate/kg/ha	Average in the whole period		Value in June 29^a^	
		E_w_/%	E_N_/%	E_w_/%	E_N_/%
Border irrigation	750	56.31	62.61	56.86	72.74
	600	56.36	62.57	55.14	67.34
	450	56.30	60.80	57.83	70.01
	300	56.33	61.51	57.67	64.90
Furrow irrigation	750	56.87	62.15	57.54	71.36
	600	56.92	62.82	56.25	63.05
	450	56.87	62.17	58.83	72.95
	300	57.08	62.44	57.97	68.04
P-Value, Analysis of variance	Fertilizer rate	0.416	0.337	0.006**	0.154
	Irrigation type	0.001**	0.283	0.023*	0.959

Note: **Significant at the 0.01 level, *Significant at the 0.05 level, ^a^The low quarter distribution uniformity after 70 mm irrigation on June 28.

## Discussions

The uniformity of water and nutrient in the field under surface irrigation is lower than that under sprinkler and drip irrigation because of the difference on the style water distributed in the field^2^. There is no special instrument in the field for distributing water under surface irrigation. The length of border or furrow under surface irrigation is a main technical factor in the layout of field engineering^3,4^, influences the uniformity of water and nutrient, and also has effect on mechanical efficiency. The length of border or furrow reaches 90 m in the study, the uniformity of soil water is approaching weak, and the results indicate moderate heterogeneity of electrical conductivity and nitrate along length of border or furrow (Fig. 2). The DU_lqW_ for border irrigation is a little larger than that for furrow irrigation, and there is no obvious effect of fertilize rate on DU_lqN_ and DU_lqN_ (Table 2). The results are the same as the preliminary reports under border or furrow length from 30 to 90 m^16–18,21^. Both border and furrow irrigation can achieve satisfactory uniformity of soil water and nutrient. Uniformity of soil and nutrient is not an important influencing factor when a proper irrigation type is determined. The flat terrains of field and the application of laser land leveling may be the reasons of high uniformity of soil water and nutrient^28,29^. Laser land leveling is an important method for surface irrigation to increase water distribution efficiency, improve irrigation uniformity, prompt water saving, and increase mechanical efficiency^28–30^, and should be promoted broad application in field crop cultivation in Huabei Plain, China, and others similar irrigation areas.

The temporal distribution of soil water is related to irrigation and precipitation over the growth period^31,32^. Only one time irrigation was carried out through the whole growth duration, the influence of irrigation type on soil water distribution was eliminated by the precipitation (Figs. 1 and 3). E_W_ for border irrigation was significantly lower than that for furrow irrigation (Table 4). The horizontal transport of soil water along the wetted perimeter of furrows after irrigation or precipitation may induce more water to be stored in the upper soil layer^33,34^. During precipitation, furrows can collect rainwater and affect water distribution in the soil and may increase E_W_. Soil EC and nitrate content gradually decreased over the growth period and significantly decreased with lower fertilizer rates (Figs. 4 and 5), and there were no significant effects of irrigation type or fertilizer rate on E_N_. Fertilizer rate is a key factor influencing nitrate dynamic in the soil, higher fertilizer rate may cause nitrate loss to deep soil, and suitable rate should be adopted^35,36^.

The same distribution characteristics were found for EC and nitrate, and the significant correlation between electrical conductivity and nitrate is found (Table 3). Same results were also found in related research^37,38^. The measurement of soil nitrate is a relatively complex task, but there are instruments that can directly and easily measure the soil’s electrical conductivity, and the regression equation between soil nitrate and EC can be used to achieve the transformation from soil electrical conductivity to soil nitrate content.

## Conclusions

Knowledge of soil water and nitrate uniformity and distribution or border or furrow systems is required to improve irrigation and fertilizer management in field crop production and to decrease environmental pollutions. Using CV variation, the heterogeneity along the length of border or furrow is weak for soil water content, is moderate for soil nitrate content, and is moderate at the soil depth of 0–20 cm and weak at the other soil layers for electrical conductivity. The average low quarter distribution uniformity was 96.34% for soil water DU_lqW_ and 79.04% for nitrate content DU_lqN_. There was a significant effect of irrigation type on DU_lqW_, and the DU_lqW_, border irrigation was 0.8% greater than that for furrow irrigation. There was a significant influence of fertilizer rate on DU_lqN_, and the DU_lqN_ was gradually increased with a decrease in fertilizer from 750 to 300 kg/ha.

The spatial and temporal distribution of soil water in the top 0–60 cm was more variable than that at a depth of 60–100 cm. There was a significant correlation between the spatial and temporal distribution of electrical conductivity and nitrate. The soil nitrate content gradually decreased through the growth period and in deeper soil.

There was a significant effect of fertilizer rate on the spatial distribution of EC and nitrate in the soil layer, and there was a decreasing trend in the soil EC and nitrate with decreasing in fertilizer rates. Irrigation types had a significant effect on the storage efficiency of water E_W,_ the E_W_ for border irrigation was 56.63%, and was 1.1% lower than that for furrow irrigation over the whole growth period. The nitrate storage efficiency was 65.47% with no significant effect of irrigation type or fertilizer rate on nitrate storage efficiency.

The uniformity of water and nitrate along the length of a border or furrow is weak to medium longer than 90 cm, which can create non-uniform conditions for crop growth. The spatial and temporal distribution analysis indicates that furrow irrigation may store slightly more water in the top 0–60 cm of soil compared to border irrigation, and may more beneficial.
